# Validation of the Korean Version of the Functioning Assessment Short Test (K-FAST) in Patients with Bipolar Disorder

**DOI:** 10.1192/j.eurpsy.2026.10706

**Published:** 2026-07-31

**Authors:** E. Lim, B.-H. Yoon, H. Kang

**Affiliations:** 1 Department of Psychiatry, Shinsegae Hyo Hospital, Kimje; 2 Department of Psychiatry, Naju National Hospital, Naju, Korea, Republic Of

## Abstract

**Introduction:**

Bipolar disorder (BD) is characterized by alternating episodes of mania/hypomania and depression, involving disturbances of mood, energy, and cognition. Lifetime prevalence estimates are 0.6% for BD-I, 0.4% for BD-II, and 1.4% for subthreshold BD. Current focus has shifted from symptom remission to functional recovery, as BD is linked to greater impairment in quality of life compared with other mood or anxiety disorders. Functional recovery depends not only on clinical remission but also on daily life functioning.

**Objectives:**

The Functioning Assessment Short Test (FAST) is a brief, disorder-specific tool for evaluating psychosocial functioning in BD, covering six domains: autonomy, occupational, cognitive, financial, interpersonal, and leisure. This study aimed to examine the validity and reliability of the Korean version of FAST (K-FAST).

**Methods:**

A total of 209 bipolar disorder patients were recruited from 14 centers in Korea. K-FAST, Young Mania Rating Scale (YMRS), Bipolar Depression Rating Scale (BDRS), Global Assessment of Functioning (GAF) and the World Health Organization Quality of Life Assessment Instrument Brief Form (WHOQOL-BREF) were administered, and psychometric analysis of the K-FAST was conducted.

**Results:**

The internal consistency (Cronbach’s alpha) of the K-FAST was 0.95. Test-retest reliability analysis showed a strong correlation between the two measures assessed at a 1-week interval (ICC = 0.97; p < 0.001). The K-FAST exhibited significant correlations with GAF (r = −0.771), WHOQOL-BREF (r = −0.326), YMRS (r = 0.509) and BDRS (r = 0.598). A strong negative correlation with GAF pointed to a reasonable degree of concurrent validity. Although the exploratory factor analysis showed four factors, the confirmatory factor analysis of questionnaires had a good fit for a six factors model (CFI = 0.925; TLI = 0.912; RMSEA = 0.078).

**Conclusions:**

The results of this study suggest that K-FAST has good psychometric properties and may be a reliable and valid tool for measurement of functioning in Korean patients with bipolar disorder. The internal consistency coefficient was high, at a Cronbach’s alpha of 0.95, for the total scale indicating item homogeneity. In this study, the concurrent validity of the K-FAST was demonstrated based on its correlations with the GAF. Concurrent validity with the GAF scale showed a highly significant negative correlation. This result indicates that patients with high functioning evaluated using K-FAST had higher scores on the GAF scale. In conclusion, the Korean version of FAST has good psychometric properties. It is a reliable and valid instrument to evaluate functional impairment in bipolar disorder. Therefore, the use of FAST in BD patients will help to assess their functioning in various domains, and in developing more individualized treatment plans.

**Disclosure of Interest:**

None Declared

